# Trends in the Age of Cigarette Smoking Initiation Among Young Adults in the US From 2002 to 2018

**DOI:** 10.1001/jamanetworkopen.2020.19022

**Published:** 2020-10-06

**Authors:** Jessica L. Barrington-Trimis, Jessica L. Braymiller, Jennifer B. Unger, Rob McConnell, Andrew Stokes, Adam M. Leventhal, James D. Sargent, Jonathan M. Samet, Renee D. Goodwin

**Affiliations:** 1 Department of Preventive Medicine, University of Southern California, Los Angeles; 2 University of Southern California Norris Comprehensive Cancer Center, Los Angeles; 3 Department of Global Health, Boston University School of Public Health, Boston, Massachusetts; 4 Department of Psychology, University of Southern California, Los Angeles; 5 Department of Pediatrics, Dartmouth Geisel School of Medicine, Hanover, New Hampshire; 6 Colorado School of Public Health, Aurora; 7 Department of Epidemiology and Biostatistics, Graduate School of Public Health and Health Policy, The City University of New York, New York; 8 Department of Epidemiology, Mailman School of Public Health, Columbia University, New York, New York

## Abstract

**Importance:**

Success in reducing the prevalence of adolescent smoking could reflect complete prevention of smoking initiation or a shift in the age of cigarette smoking initiation from adolescence into early adulthood.

**Objective:**

To assess trends in early adult (ages 18-23 years) vs adolescent (age <18 years) cigarette smoking initiation and transition to daily cigarette smoking from 2002 to 2018.

**Design, Setting, and Participants:**

Ages at initiation of smoking and the transition to daily smoking were ascertained from the National Survey on Drug Use and Health (2002-2018), an annual, population-based, repeated cross-sectional study representative of the US population. This cross-sectional analysis was restricted to young adults who completed the survey at ages 22 to 23 years during survey years 2002 to 2018 to limit potential age-related recall bias. Retrospectively collected age of cigarette smoking initiation was assessed among ever cigarette smokers; age of transition to daily smoking was assessed among ever daily cigarette smokers. Data analysis was performed from June 2019 to July 2020.

**Exposures:**

Calendar year of survey (2002 to 2018).

**Main Outcomes and Measures:**

The main outcomes were population-weighted cigarette smoking prevalence and cigarette smoking initiation and transition to daily smoking in adolescence (age <18 years) vs early adulthood (ages 18-23 years).

**Results:**

Among 71 756 young adults aged 22 to 23 years (38 226 women [50.5%]), ever cigarette smoking prevalence decreased from a population-weighted estimate of 74.6% (95% CI, 73.1%-75.9%) in 2002 to 51.4% (95% CI, 49.3%-53.5%) in 2018 (*P* < .001). Daily smoking prevalence rates similarly decreased from 41.1% (95% CI, 39.1%-43.1%) in 2002 to 20.2% (95% CI, 18.6%-21.8%) in 2018 (*P* < .001). However, among 48 015 ever smokers, the proportion initiating smoking in early adulthood (ages 18-23 years) increased over this time, from 20.6% (95% CI, 18.5%-22.8%) in 2002 to 42.6% (95% CI, 39.6%-45.7%) in 2018 (*P* < .001). Similarly, among 24 490 daily cigarette smokers, the proportion who transitioned to daily smoking in early adulthood increased from 38.7% (95% CI, 35.9%-41.6%) in 2002 to 55.9% (95% CI, 52.0%-59.8%) in 2018 (*P* < .001).

**Conclusions and Relevance:**

A substantial proportion of beginning smokers and most new daily smokers are now young adults, reflecting a shift from adolescence to early adulthood, a population segment once considered beyond the critical risk period for cigarette smoking onset. Expanding the long-standing emphasis on adolescent surveillance and prevention in adolescence to include the young adult population is warranted.

## Key Points

**Question:**

Has the proportion of cigarette smokers initiating smoking in early adulthood (ages 18-23 years) vs adolescence (age <18 years) increased from 2002 to 2018?

**Findings:**

In this repeated cross-sectional study including 71 756 young adults aged 22 to 23 years, the proportion of ever smokers who initiated cigarette smoking in early adulthood more than doubled between 2002 and 2018, and the proportion of daily cigarette smokers who transitioned to daily smoking in early adulthood also increased from 38.7% in 2002 to 55.9% in 2018.

**Meaning:**

These findings suggest that smoking prevention efforts typically targeting adolescents should be expanded to address young adults, who account for an increasing and substantial proportion of new smokers.

## Teaser

This cross-sectional study assesses trends in early adult (ages 18-23 years) vs adolescent (age <18 years) cigarette smoking initiation and transition to daily cigarette smoking from 2002 to 2018.

## Introduction

Smoking continues to have a substantial adverse impact on the public health of the US population, with current estimates of more than 480 000 deaths in the US attributable to smoking and secondhand smoke exposure annually.^1^ Historically, most adult smokers in the US began smoking before age 18 years.^1,2^ Among adult smokers aged 30 to 39 years in the US in 2012, 90% reported initiating cigarette use at or before the age of 18 years,^1^ reflecting the peak rates of adolescent cigarette smoking in the early to mid-1990s when this cohort was in adolescence.^3^ Consequently, most tobacco prevention efforts—including age restrictions, media campaigns, and educational materials—have targeted middle school and high school students.^4^ These efforts have successfully reduced population smoking prevalence among adolescents; daily cigarette smoking among 12th grade students in the US declined from its most recent peak of 24.6% in 1997 to 3.6% in 2018.^3^ Daily cigarette smoking among those younger than 18 years is at an all-time low, portending a decline in tobacco-related death and disease. Yet, this decrease in youth smoking may be misleading or overly optimistic if an appreciable proportion of smokers now initiate or transition to daily smoking in early adulthood, particularly if that proportion is increasing over time.

Continued efforts to reduce the prevalence of cigarette smoking and, in particular, to stop the transition to daily smoking remain critical. One key target group is the young adult population, among whom cigarette smoking has only incrementally declined over the last decade. A recent study^5^ using the same national survey used here (the National Survey on Drug Use and Health [NSDUH]) found that past-year initiation of any cigarette use among participants aged 18 to 21 years increased from 2002 to 2009 and then decreased through 2015, whereas past-year initiation of any cigarette use among participants aged 22 to 25 years did not change over this study period. However, the study^5^ did not assess the relative proportions of those initiating in adolescence or in adulthood among the young adult smokers in the sample. A shift toward a later age of smoking initiation would have substantial implications for targeted prevention efforts to reduce tobacco-related morbidity and mortality. An increase in the relative proportion of smokers who initiate cigarette smoking in early adulthood (vs adolescence) would document the positive impact of tobacco control in delaying initiation beyond adolescence, but also suggests that such efforts delayed but did not immunize youth from initiation of cigarette smoking.

We investigated trends in the age of any cigarette smoking initiation and transition to daily cigarette smoking using data from young adults aged 22 to 23 years who participated in the NSDUH between 2002 and 2018. The age group 22 to 23 years was selected to examine young adult cigarette smoking while limiting the potential for recall bias across all years of the study (ie, recall of the age of smoking initiation is likely more accurate for younger adults aged 22 to 23 years vs older adults who may have started smoking decades earlier) and because most initiation (approximately 97%) has historically occurred before the age of 23 years.^1^

## Methods

### Participants

The current cross-sectional study includes data from 71 756 participants in the NSDUH who participated in any year between 2002 and 2018, who were aged 22 or 23 years at the time of survey, and who provided data on ever smoking (as discussed later in this article). Data collection procedures for the NSDUH are described elsewhere.^6^ Briefly, the NSDUH is a nationally representative, annual, cross-sectional survey of tobacco, alcohol, and drug use, and of other health-related issues in the US.^7^ NSDUH uses a multistage, stratified, complex clustering method to select a new sample of participants each year from all 50 states in the US and from the District of Columbia. Data were collected using computer-assisted personal interviewing methods in the homes of participants. Data collection for the NSDUH was approved by the National Center for Health Statistics Research Ethics Review Board. All participants provided verbal informed consent before data collection. Analysis of deidentified data from the survey is exempt from the federal regulations for the protection of human research participants in accordance with 45 CFR §46. Analysis of restricted data through the National Center for Health Statistics Research Data Center is also approved by the National Center for Health Statistics Research Ethics Review Board. This study follows the Strengthening the Reporting of Observational Studies in Epidemiology (STROBE) reporting guideline for observational studies.

### Measures

#### Ever Cigarette Smoking

Participants were asked whether they had ever smoked all or part of a cigarette; participants who responded yes were considered ever smokers. If participants responded affirmatively, they were asked to provide the age at which they first smoked all or part of a cigarette (age of smoking initiation, in years). Primary analyses evaluated age of initiation of any cigarette use; in sensitivity analyses, we evaluated the age of initiation among a sample restricted to those who had smoked at least 100 cigarettes in their lifetime, a widely used criterion for being an ever smoker.^1^

#### Daily Cigarette Smoking

Participants who had ever smoked a cigarette were also asked whether there had ever been a period in their life when they smoked cigarettes every day for at least 30 days. Those responding yes (ever daily cigarette smokers) were asked to provide the age at which they first started smoking cigarettes every day (age of transition to daily smoking, in years).

### Demographic Characteristics

Participants self-reported gender (male or female), race/ethnicity (non-Hispanic [NH] White, NH Black or African American, NH Native American or Alaska Native, NH Native Hawaiian or other Pacific Islander, NH Asian, NH >1 race, or Hispanic), highest level of education (some high school or less, high school diploma or general educational diploma, some college, or college degree or higher), annual family income (<$20 000, $20 000-$49,999, $50 000-$74 999, or ≥$75 000), and marital status (never married, married, divorced or separated, or widowed).

### Statistical Analysis

We report population-standardized prevalence estimates for categorized outcomes of interest (eg, early adult [ages 18-23 years] vs adolescent [<18 years] initiation of ever smoking or transition to daily smoking) and population-standardized means for continuous outcomes of interest (eg, age of any smoking initiation and age of transition to daily smoking), by survey year. Prevalence estimates of the rates of ever smoking and daily smoking used the whole sample. Analyses examining the proportion of smokers who initiated in early adulthood and mean age of any smoking initiation were restricted to 48 015 ever cigarette smokers. Analyses examining the proportion of smokers who transitioned to daily cigarette smoking in early adulthood and mean age of transition to daily smoking were restricted to 24 490 participants who reported having ever smoked daily for 30 or more days. Differences in the prevalence of ever smoking by year were assessed using logistic regression models adjusting for gender, race/ethnicity, income, education level, and marital status, variables that were selected a priori on the basis of prior associations with smoking^1^; similar models were used to assess differences in ever daily smoking by year. Logistic regression models adjusting for the same covariates were also used to assess differences in the proportion of ever smokers who initiated in early adulthood (ages 18-23 years) vs adolescence (age <18 years) by year in the sample restricted to ever smokers; in a separate model, differences in the proportion of smokers who transitioned to daily smoking in early adulthood vs adolescence in the sample were restricted to ever daily smokers. Differences in the mean age of initiation of ever smoking and mean age of transition to daily smoking by year were assessed using linear regression models adjusting for the same covariates. All analyses were 2-tailed, with α = .05 denoting statistical significance. Analyses were conducted using Stata statistical software version 15.1 (StataCorp) and used survey weighting procedures (ie, using the svy suite of commands in Stata) based on NSDUH sampling weights to account for any differences in the sample from year to year; the NSDUH supplied imputed values for any missing data.^8^ Data analysis was performed from June 2019 to July 2020.

### Sensitivity Analyses

To assess the robustness of the results reported here, we conducted a series of sensitivity analyses. First, we restricted the sample to participants who had smoked at least 100 cigarettes in their lifetime, which is a commonly used metric for classification as a smoker.^1^ Second, we reran all analyses among participants of different ages at survey completion (ie, among those who were aged 24-25 years at survey completion, and among those who were aged 21 years at survey completion) to assess whether any differences in patterns over time were observed for different age groups. Finally, we conducted a similar analysis using data from the National Health Interview Survey (NHIS) (see the eAppendix in the Supplement).

## Results

### Demographic Characteristics

The analytic sample included 71 756 participants aged 22 to 23 years at survey completion in a survey year from 2002 to 2018 (Table). After population standardization, the sample was approximately evenly distributed by gender (38 226 women [50.5%]), and most participants were NH White (42 619 participants [58.9%]). Annual family income was less than $50 000 for most participants (<$20 000, 24 230 participants [31.7%]; $20 000-$49 999, 27 238 participants [36.1%]). Most participants (58 065 [83.5%]) had never been married. The composition of the sample was generally similar across years, although the proportion of the sample that was NH White declined in more recent years, whereas the proportion of Asian and Hispanic participants increased. In addition, in recent years participants were more educated, had higher annual family income, and more participants were not married at the time of survey (see eTable 1 in the Supplement for demographic distribution of the sample by year).

**Table.** Demographic Characteristics of Participants Included in the Analytic Sample Among Those Aged 22 to 23 Years at Survey Completion, Aggregated Across 2002 to 2018

Characteristic	Participants, unweighted No. (weighted %)		
	Total (N = 71 756)	Ever smokers (n = 48 015)	Ever daily smokers (n = 24 490)
Gender			
Male	33 530 (49.5)	23 907 (53.1)	12 175 (53.1)
Female	38 226 (50.5)	24 108 (46.9)	12 315 (46.9)
Race/ethnicity			
NH White	42 619 (58.9)	31 074 (65.0)	17 340 (72.5)
NH Black	9633 (13.8)	4918 (10.6)	2174 (9.1)
NH Native American or Alaska Native	1140 (0.7)	923 (0.8)	522 (0.8)
NH Hawaiian or Pacific Islander	443 (0.5)	288 (0.5)	160 (0.5)
NH Asian	3034 (5.2)	1493 (3.8)	530 (2.8)
NH multiple races	2218 (1.6)	1595 (1.7)	911 (1.8)
Hispanic	12 669 (19.3)	7724 (17.7)	2853 (12.5)
Highest level of education			
Less than high school	9743 (12.3)	7273 (13.8)	5137 (19.7)
High school diploma or general educational diploma	21 336 (28.2)	15 249 (30.3)	9430 (37.3)
Some college	24 812 (35.5)	16 250 (35.0)	7510 (32.2)
College degree or higher	15 865 (24.0)	9243 (20.9)	2413 (10.8)
Annual family income, $			
<20 000	24 230 (31.7)	16 013 (32.0)	8333 (32.0)
20 000-49 999	27 238 (36.1)	18 666 (36.2)	9990 (39.5)
50 000-74 999	8878 (12.8)	5896 (12.9)	2919 (12.3)
≥75 000	11 410 (19.3)	7440 (18.9)	3248 (16.3)
Marital status			
Never married	58 065 (83.5)	55 166 (83.3)	19 425 (81.5)
Married	11 840 (14.3)	11 383 (14.5)	4008 (14.7)
Divorced or separated	1726 (2.0)	1670 (2.1)	986 (3.6)
Widowed	125 (0.2)	125 (0.2)	71 (0.3)

Abbreviation: NH, non-Hispanic.

### Population Trends in Prevalence of Ever Smoking and Daily Smoking

From 2002 to 2018, the prevalence of ever smoking among participants aged 22 to 23 years decreased from 74.6% (95% CI, 73.1%-75.9%) to 51.4% (95% CI, 49.3%-53.5%) (*P* < .001 for test of difference in prevalence for 2002 vs 2018) (eFigure 1 in the Supplement). Over this period, the prevalence of ever daily cigarette smoking also decreased substantially, from 41.1% (95% CI, 39.1%-43.1%) in 2002 to 20.2% (95% CI, 18.6%-21.8%) in 2018 (*P* < .001 for test of difference in prevalence for 2002 vs 2018).

### Trends in Young Adult Onset of Cigarette Smoking Over Time Among Ever Smokers

**Figure 1.**
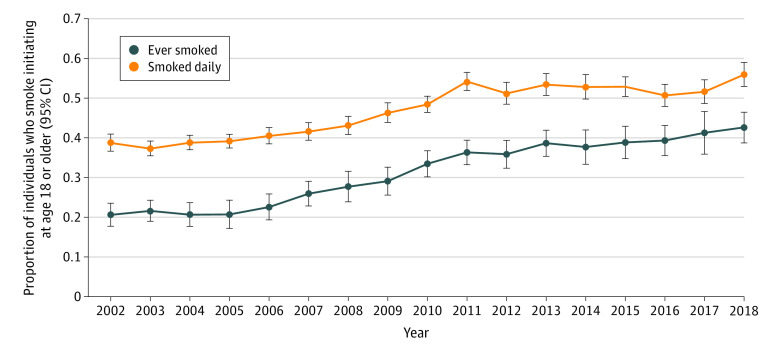
Proportion of Ever Cigarette Smokers Who Began Smoking at Ages 18 to 23 Years and Proportion of Ever Daily Smokers Who Began Smoking Daily at Ages 18 to 23 years by Year From 2002 to 2018, Among Participants Aged 22 to 23 Years at Survey Completion Error bars denote 95% CIs.

**Figure 2.**
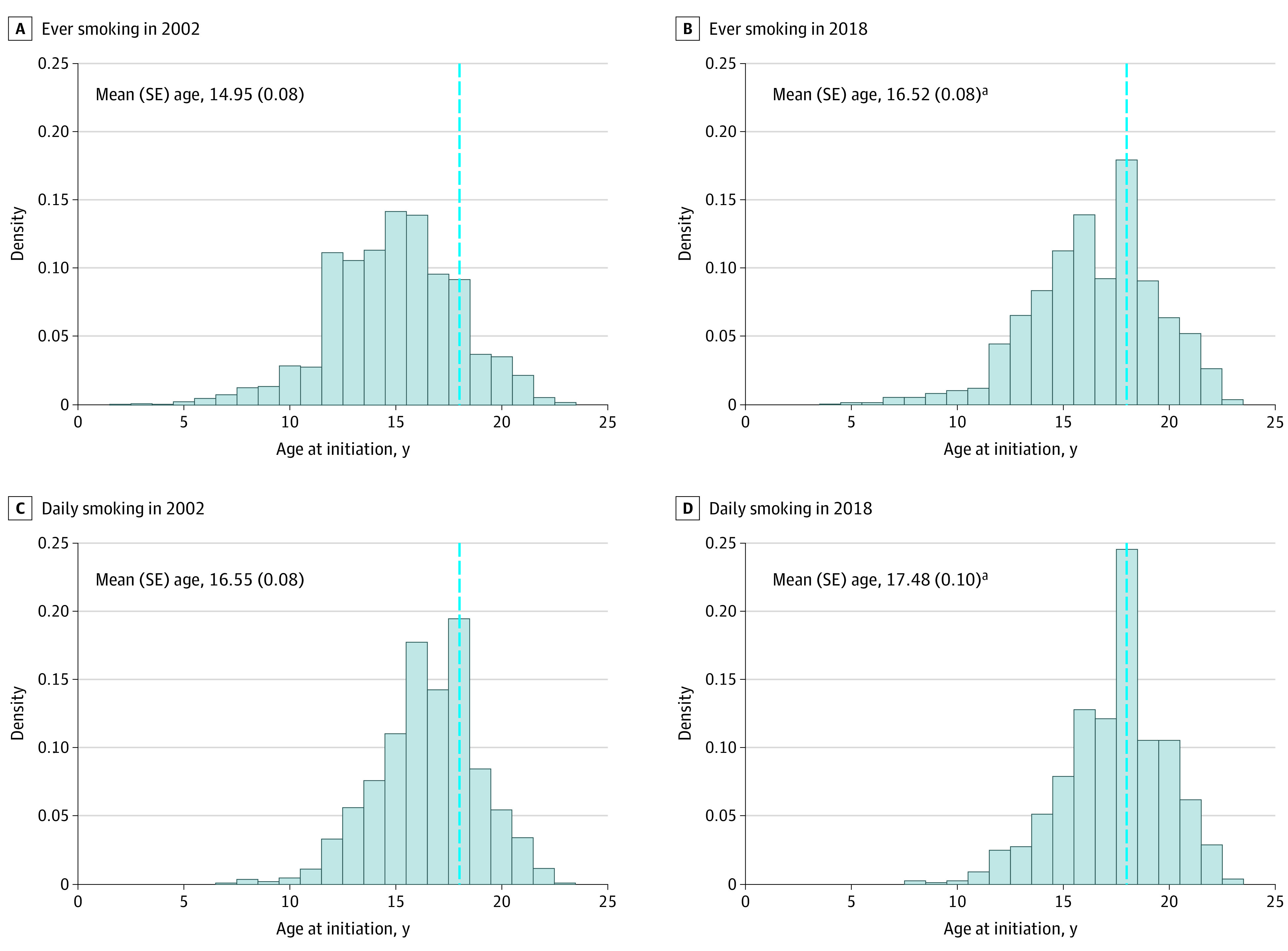
Distribution of Age at Initiation Among Ever Cigarette Smokers in 2002 and 2018 and Age of Initiation of Daily Smoking Among Daily Smokers in 2002 and 2018 Among Participants Aged 22 to 23 Years at Survey Administration Panels A and B show data for ever smokers in 2002 (A) and 2018 (B). Panels C and D show data for daily smokers in 2002 (C) and 2018 (D). Dashed lines indicate smoking initiation at age 18 years. ^a^*P* < .001 for test of difference in mean age of initiation for 2002 vs 2018, adjusting for gender, race/ethnicity, education level, income, and marital status.

The proportion of young adult ever smokers initiating smoking in early adulthood (ages 18-23 years) more than doubled from 20.6% (95% CI, 18.5%-22.8%) in 2002 to 42.6% (95% CI, 39.6%-45.7%) in 2018 (*P* < .001 for test of difference in proportion for 2002 vs 2018) (Figure 1). Examination of the continuous age of smoking initiation outcome over time revealed that over this period, the distribution of the age of smoking initiation shifted overall toward older ages in more recent years (Figures 2A and B and eTable 2 in the Supplement). In 2002, the peak frequency of age of initiation was 15 years, with a mean (standard error [SE]) age of initiation, of 14.95 (0.08) years, and was normally distributed (Figure 2A). In 2018, the distribution of age of initiation was somewhat bimodal, with a minor peak at age 16, and a larger peak at age 18 years (Figure 2B). The first year during which the peak age of initiation was 18 years was 2014; from 2015 to 2018, the age 18 years frequency peak further increased over time (see eFigure 2A in the Supplement). The mean (SE) age of initiation in 2018 was 16.52 (0.08) years, statistically significantly higher than the mean (SE) age of initiation in 2002, 14.95 (0.08) years (*P* < .001).

### Trends in Young Adult Transition to Daily Cigarette Smoking Over Time Among Ever Daily Smokers

The proportion of ever daily cigarette smokers who first transitioned to daily smoking in early adulthood (age ≥18 years) also increased over the study period, from 38.7% (95% CI, 35.9%-41.6%) in 2002 to 55.9% (95% CI, 52.0%-59.8%) in 2018 (*P* < .001 for test of difference in proportion for 2002 vs 2018) (Figure 1). When examined as a continuous outcome, the distribution of the age at which smokers initiated daily smoking overall also shifted toward older ages from 2002 to 2018 (Figures 2C and D and eTable 3 in the Supplement). In 2002, a bimodal distribution was observed for age of transition to daily smoking, with a minor peak observed at age 16, and slightly higher peak observed at age 18 (Figure 2C); the mean (SE) age of transition to daily smoking was 16.55 (0.08) years. In 2018, a unimodal distribution of age of transition to daily smoking was observed, with a large single peak observed at age 18 years (Figure 2D). The mean (SE) age of transition to daily smoking in 2018 was 17.48 (0.10) years, which was significantly higher than the mean age of transition to daily smoking in 2002, 16.55 (0.08) years (*P* < .001).

### Sensitivity Analyses

#### Restriction to Those Who Had Smoked at Least 100 Cigarettes

Patterns of young adult cigarette smoking initiation over time similarly increased from 2002 to 2018 after restricting the sample to ever smokers who had smoked at least 100 cigarettes in their lifetime, although a smaller proportion overall began smoking in early adulthood (eTable 2 and eTable 3 in the Supplement). For example, in 2002, 12.1% of these more established smokers began smoking in early adulthood; in 2018, 25.9% of smokers had begun smoking in early adulthood (eTable 2 in the Supplement). Among ever daily smokers, patterns across time and by year were similar after restricting to those who had ever smoked 100 cigarettes, with 37.8% beginning to smoke daily in early adulthood in 2002, and 54.5% beginning to smoke daily in early adulthood in 2018 (eTable 3 in the Supplement).

#### Trends Among Those Aged 21 or 24 to 25 Years at Survey Completion

Similar results were observed in samples of those who were aged 21 years, or, in separate models, aged 24 to 25 years, at survey completion (eFigure 3 in the Supplement). For example, among those aged 21 years, in 2002, 16.3% of ever smokers began smoking at 18 or older, whereas in 2018, 42.6% began smoking at age 18 years or older (eFigure 3A in the Supplement). Among those aged 24 to 25 years, in 2002, 25.3% began smoking in early adulthood, whereas in 2018, 43.3% began smoking in early adulthood (eFigure 3B in the Supplement). Among both age groups (age 21 and age 24-25 years), transition to daily smoking in early adulthood (vs adolescence) followed similar patterns.

#### Trends in Age of Regular Smoking Initiation Among Participants in the NHIS

Findings using data from the NHIS were similar (eAppendix in the Supplement). Survey participants were asked at what age they started smoking regularly. The frequency that constitutes regular smoking is not further defined by the survey. From 2002 to 2018, the proportion of regular smokers who began smoking regularly in early adulthood increased. In 2002, 33.3% (95% CI, 28.6%-38.4%) of regular smokers initiated regular smoking in early adulthood, compared with 51.3% (95% CI, 41.5%-61.0%) in 2018 (*P* = .01) (eFigure 4 in the Supplement). Among lifetime regular smokers in the NHIS, we found an increase in the mean (SE) age of regular smoking initiation that was not statistically significant, from 16.33 (0.14) years in 2002 to 17.00 (0.25) in 2018 (*P* = .13).

## Discussion

The prevalence of ever and of daily cigarette smoking decreased among those aged 22 to 23 years over the period from 2002 to 2018. These changes may reflect decades of continued tobacco control policy and prevention work,^2^ along with recent, more concerted tobacco control efforts directed at youth.^9^ Yet, from 2002 to 2018, the proportion of cigarette smokers who initiated any cigarette smoking and transitioned to daily cigarette smoking in early adulthood (at age ≥18 years) increased substantially. These findings may reflect delayed onset rather than absolute prevention of cigarette initiation. Thus, the long-standing focus of prevention efforts in middle and high schools and regulatory efforts targeted to youth younger than 18 years may be insufficient to prevent the adverse consequences of cigarette smoking. Prevention efforts in the young adult population are needed.

Historically, later age of cigarette smoking initiation has been strongly associated with lower risk of lifelong smoking, nicotine dependence, respiratory and cardiovascular health effects, and other adverse effects of cigarette use.^1,2^ Findings from the current study showing a later age of cigarette smoking initiation suggest positive life course effects in reducing the overall magnitude of adverse smoking-related health outcomes.^1^ Yet, the finding that more than 40% of ever smokers began to smoke and more than 50% of daily smokers began to smoke daily in early adulthood means that efforts to date may have lessened the severity of long-term effects of cigarette smoking without eliminating tobacco-related health consequences for those who age into early adulthood without yet having begun to smoke.

Recent nationwide efforts to prevent cigarette smoking initiation have largely targeted youth, neglecting the vulnerable young adult population. For example, the Real Cost Campaign, a successful antitobacco campaign by the US Food and Drug Administration’s Center for Tobacco Products, targets youth aged 12 to 17 years.^10^ Other tobacco control prevention efforts (eg, education campaigns) have been primarily focused on middle and high school students.^1,2,11,12,13^ Because an increasingly large proportion of cigarette smokers are initiating smoking and transitioning to daily smoking in early adulthood, prevention efforts targeted to young adults are needed to continue to reduce smoking initiation rates and subsequent reductions in tobacco-related morbidity and mortality. Notably, the Truth Campaign relaunched their campaign in 2014 to target individuals aged 15 to 21 years.^14^ Other prevention campaigns have specifically targeted college students in this age group; yet, smoking rates are higher among young adults not currently enrolled in college,^15^ a hard-to-reach priority group that could benefit from focused prevention efforts. Efforts to prevent escalation in smoking after initiation,^16^ or to encourage cessation among smokers early in their smoking trajectory,^17^ are also needed to prevent transition to daily, established smoking and the development of nicotine dependence.

As with prevention campaigns and educational efforts, regulatory policies have also historically been focused on youth; a shift in policies that target the young adult population may further reduce the adverse public health impact of observed trends in young adult smoking initiation. For example, until recently,^18^ the sale of tobacco products was illegal only for those younger than age 18 years. The implementation of Tobacco 21 policies, which raise the minimum legal age of the sale of tobacco products to those aged 21 years or older (and which were recently implemented across the US in late December 2019),^19,20^ could have a substantial impact on reducing cigarette smoking initiation among young adults by continuing to delay the mean age of initiation into later adulthood, or, ideally, preventing initiation altogether.^21,22,23^ Such efforts could also discourage continued smoking among those who have already initiated, thus preventing escalation in smoking frequency.^16^ Enforcement efforts are needed to ensure that Tobacco 21 policies have maximal impact on reducing young adult initiation.^21^ A recent study^24^ found high rates of noncompliance with California’s Tobacco 21 law; a substantial proportion of retailers sold tobacco products to underaged young adults (aged 18-20 years) who attempted to purchase tobacco products as part of that study. Other regulatory efforts to reduce smoking initiation that may not be specifically targeted to the youth (or young adult) population may also exert a continued influence on preventing initiation or preventing transition to daily smoking among both youth and young adults. For example, comprehensive tobacco control policies that mandate smoke-free public places, excise taxes on cigarettes (and other tobacco products) at the local, state, and federal levels; nontax price increases (including minimum price, minimum pack size, and restrictions on price promotions); general mass media campaigns; and tobacco retailer licensing with sufficient fees to cover enforcement efforts may all reduce both adolescent and young adult smoking^2^ and should continue to be implemented widely.

The increase in the proportion of smokers transitioning to daily smoking in early adulthood leveled off from 2013 to 2017, although an increase was observed from 2017 to 2018. It is possible that the initial surge in use of electronic cigarettes (e-cigarettes) among US young adults around that time^15^ stalled the trend toward initiation in young adulthood up to that point. If e-cigarettes draw new youth into early nicotine use,^25,26^ and e-cigarette users then are more likely to begin smoking cigarettes,^27,28,29,30,31,32,33,34,35,36,37,38,39,40,41,42,43,44^ it is possible that e-cigarettes may result in greater cigarette initiation in adolescence than would otherwise be expected (ie, without e-cigarettes, individuals might have begun to smoke later, in early adulthood, but e-cigarettes may speed up the initiation for some proportion of the population). Alternatively, e-cigarettes may result in a transition away from cigarettes before individuals become daily smokers, or a delay in progression to daily smoking until later in adulthood. Because we did not have data on e-cigarette use or age of e-cigarette initiation (or data on which product was used first) in the survey years included in the current study, we were unable to evaluate the impact of e-cigarettes on trends in the age of initiation.

Although the operational definition of more established smoking patterns differed between NSDUH and NHIS (ie, age of transition to daily smoking vs regular smoking), trends in the age of transition to daily or regular smoking across the 2 studies over the same period were remarkably similar, highlighting the robustness of our findings. In both studies, more than one-half of established smokers (daily or regular smokers) began patterns of established smoking in early adulthood, not adolescence.

### Limitations

Several limitations of this study are noted. The findings are based on self-reported measures collected at ages 22 to 23 years. The reported age of ever cigarette use and daily cigarette use is, therefore, subject to recall bias. However, any recall bias among those of the same age group (aged 22-23 years at data collection) in each survey year from 2002 to 2018 is likely consistent over the period of the study and thus is unlikely to influence the temporal trends observed within this study (ie, any recall bias is likely to be nondifferential by survey year). The presented prevalence estimates of age of initiation by survey year are weighted^8^ but are not further adjusted for other sociodemographic, environmental, or behavioral risk factors that may have changed over time. Despite these limitations, the public health significance of increases in the proportion of ever and daily cigarette smokers initiating in early adulthood remains and is an imperative for action.

## Conclusions

In this repeated cross-sectional study of US young adults, the proportion of cigarette smokers initiating cigarette use in early adulthood (vs adolescence) increased from 2002 to 2018. Although historically, most smokers began to smoke before age 18 years, in recent years, an appreciable proportion of smokers started smoking in early adulthood. The definition of early-onset smoking may need to be reconsidered to include a developmental phase once thought to be beyond the peak risk period for cigarette onset, but, as with many other aspects of adolescence, seem to be extending beyond the original parameters of prior generations. The expansion of effective nationwide smoking prevention programs and regulatory policies beyond adolescence to additionally target the young adult population warrant consideration to successfully prevent smoking initiation in the US.

## Notes

Supplement. **eAppendix.** Supplemental Methods **eTable 1.** Demographic Characteristics of Participants Included in the Analytic Sample Among Those Aged 22-23 At Survey Completion, by Year, From 2002-2018 **eTable 2. **Cumulative Proportion of Participants Who Had Ever Initiated Cigarette Smoking, by Each Age of Initiation and Year (2002-2018), Among Ever Smokers and Among Ever Smokers Who Had Smoked More Than 100 Cigarettes, for Survey Respondents Aged 22-23 at Survey Completion **eTable 3. **Cumulative Proportion of Participants Who Had Ever Smoked Daily, by Each Age of First Daily Cigarette Smoking and Year (2002-2018), Among Ever Daily Smokers and Among Ever Daily Smokers Who Had Smoked More Than 100 Cigarettes for Survey Respondents Aged 22-23 at Survey Completion **eFigure 1. **Prevalence (95% CI) of Ever Smoking, and Daily Smoking by Year From 2002-2018, Among Survey Participants Aged 22-23 **eFigure 2. **Distribution of (A) Age at Initiation Among Ever Cigarette Smokers and (B) Age at Transition to Daily Smoking Among Ever Daily Smokers, by Year, Among Participants Aged 22-23 at Survey Administration **eFigure 3. **Proportion (95% CI) of Ever Cigarette Smokers Who Began Smoking at Age 18 or Older, and Proportion of Ever Daily Smokers Who Began Smoking Daily at Age 18 or Older, by Year From 2002-2018, Among Participants (A) Aged 21 and (B) Aged 24-25 at Survey Completion **eFigure 4. **Proportion (95% CI) of Regular Cigarette Smokers Who Began Smoking at Age 18 or Older in the NHIS Study, by Year From 2002-2018, Among Participants Aged 22-23 at Survey Completion

## References

[1] Centers for Disease Control and Prevention 2014 Surgeon General’s report: the health consequences of smoking—50 years of progress. Published 2014 Accessed September 3, 2020. https://www.cdc.gov/tobacco/data_statistics/sgr/50th-anniversary/index.htm

[2] Centers for Disease Control and Prevention Preventing Tobacco Use Among Youth and Young Adults: A Report of the Surgeon General. Centers for Disease Control and Prevention, Office on Smoking and Health; 2012. 22876391

[3] Miech R, Johnston L, O'Malley P, Bachman J, Schulenberg J, Patrick M Monitoring the future: national survey results on drug use, 1975-2019—volume 1, secondary school students. Published 2019 Accessed September 3, 2020. http://www.monitoringthefuture.org/pubs/monographs/mtf-vol1_2019.pdf

[4] Das JK, Salam RA, Arshad A, Finkelstein Y, Bhutta ZA Interventions for adolescent substance abuse: an overview of systematic reviews. J Adolesc Health. 2016;59(4)(suppl):S61-S75. doi:10.1016/j.jadohealth.2016.06.021 27664597 PMC5026681

[5] Cantrell J, Bennett M, Mowery P, Patterns in first and daily cigarette initiation among youth and young adults from 2002 to 2015. PLoS One. 2018;13(8):e0200827. doi:10.1371/journal.pone.0200827 30096141 PMC6086419

[6] Bose J, Hedden SL, Lipari RN, Park-Lee E Key substance use and mental health indicators in the United States: results from the 2017 national survey on drug use and health. Published September 2018 Accessed September 3, 2020. https://www.samhsa.gov/data/sites/default/files/cbhsq-reports/NSDUHFFR2017/NSDUHFFR2017.pdf

[7] Substance Abuse and Mental Health Services Administration National survey on drug use and health. Updated January 1, 2020 Accessed September 3, 2020. https://nsduhweb.rti.org/respweb/homepage.cfm

[8] Substance Abuse and Mental Health Services Administration; Center for Behavioral Health Statistics and Quality 2017 National survey on drug use and health: public use file codebook. Published October 23, 2018 Accessed September 3, 2020. https://samhda.s3-us-gov-west-1.amazonaws.com/s3fs-public/field-uploads-protected/studies/NSDUH-2017/NSDUH-2017-datasets/NSDUH-2017-DS0001/NSDUH-2017-DS0001-info/NSDUH-2017-DS0001-info-codebook.pdf

[9] Lundeen RE Jr Tobacco under the FDA: a summary of the Family Smoking Prevention and Tobacco Control Act. Health Care Law Mon. 2009;2009(9):2-9. 19771935

[10] Santiago S, Talbert EC, Benoza G Finding Pete and Nikki: defining the target audience for “the real cost” campaign. Am J Prev Med. 2019;56(2)(suppl 1):S9-S15. doi:10.1016/j.amepre.2018.07.040 30661530

[11] Truth Initiative Truth initiative: inspiring tobacco-free lives. Published 2019 Accessed June 1, 2019. http://www.truthinitiative.org

[12] US Food and Drug Administration This free life campaign. Published 2018 Accessed June 1, 2019. https://www.fda.gov/tobacco-products/public-health-education-campaigns/free-life-campaign

[13] US Food and Drug Administration Fresh empire campaign. Published 2018 Accessed June 1, 2019. https://www.fda.gov/TobaccoProducts/PublicHealthEducation/PublicEducationCampaigns/FreshEmpireCampaign/default.htm

[14] Mann Global Health Case study: Truth campaign. Accessed September 10, 2020. https://mannglobalhealth.com/microsite/downloads/MGH-case-study-truth.pdf

[15] Schulenberg J, Johnston L, O'Malley P, Bachman J, Miech R, Patrick M Monitoring the future: national survey results on drug use, 1975-2019—volume II, college students and adults ages 19-60. Published 2019 Accessed September 3, 2020. http://www.monitoringthefuture.org//pubs/monographs/mtf-vol2_2019.pdf

[16] Villanti AC, Niaura RS, Abrams DB, Mermelstein R Preventing smoking progression in young adults: the concept of prevescalation. Prev Sci. 2019;20(3):377-384. doi:10.1007/s11121-018-0880-y 29525899 PMC6131072

[17] Villanti AC, West JC, Klemperer EM, Smoking-cessation interventions for U.S. young adults: updated systematic review. Am J Prev Med. 2020;59(1):123-136. doi:10.1016/j.amepre.2020.01.021 32418800 PMC7453837

[18] US Food and Drug Administration Retail sales of tobacco products. Updated June 3, 2020 Accessed January 2, 2020. https://www.fda.gov/tobacco-products/compliance-enforcement-training/retail-sales-tobacco-products

[19] Campaign for Tobacco Free Kids States and localities that have raised the minimum legal sale age for tobacco products to 21. Published 2019 Accessed September 3, 2020. https://www.tobaccofreekids.org/assets/content/what_we_do/state_local_issues/sales_21/states_localities_MLSA_21.pdf

[20] US Food and Drug Administration Selling tobacco products in retail stores. Published 2019 Accessed September 3, 2020. https://www.fda.gov/tobacco-products/retail-sales-tobacco-products/selling-tobacco-products-retail-stores

[21] Bonnie RJ, Stratton K, Kwan LY, eds. Public Health Implications of Raising the Minimum Age of Legal Access to Tobacco Products. The National Academies Press; 2015. doi:10.17226/18997 26269869

[22] Macinko J, Silver D Impact of New York City’s 2014 increased minimum legal purchase age on youth tobacco use. Am J Public Health. 2018;108(5):669-675. doi:10.2105/AJPH.2018.304340 29565664 PMC5888055

[23] Kessel Schneider S, Buka SL, Dash K, Winickoff JP, O’Donnell L Community reductions in youth smoking after raising the minimum tobacco sales age to 21. Tob Control. 2016;25(3):355-359. doi:10.1136/tobaccocontrol-2014-052207 26071428

[24] Roeseler A, Vuong TD, Henriksen L, Zhang X Assessment of underage sales violations in tobacco stores and vape shops. JAMA Pediatr. 2019;173(8):795-797. doi:10.1001/jamapediatrics.2019.1571 31233124 PMC6593621

[25] Barrington-Trimis JL, Urman R, Leventhal AM, E-cigarettes, cigarettes, and the prevalence of adolescent tobacco use. Pediatrics. 2016;138(2):e20153983. doi:10.1542/peds.2015-3983 27401102 PMC4960723

[26] Dutra LM, Glantz SA E-cigarettes and national adolescent cigarette use: 2004-2014. Pediatrics. 2017;139(2):e20162450. doi:10.1542/peds.2016-2450 28115540 PMC5260150

[27] Soneji S, Barrington-Trimis J, Wills T, Association between initial use of e-cigarettes and subsequent cigarette smoking among adolescents and young adults: a systematic review and meta-analysis. JAMA Pediatr. 2017;171(8):788-797. doi:10.1001/jamapediatrics.2017.1488 28654986 PMC5656237

[28] Leventhal AM, Strong DR, Kirkpatrick MG, Association of electronic cigarette use with initiation of combustible tobacco product smoking in early adolescence. JAMA. 2015;314(7):700-707. doi:10.1001/jama.2015.8950 26284721 PMC4771179

[29] Primack BA, Soneji S, Stoolmiller M, Fine MJ, Sargent JD Progression to traditional cigarette smoking after electronic cigarette use among US adolescents and young adults. JAMA Pediatr. 2015;169(11):1018-1023. doi:10.1001/jamapediatrics.2015.1742 26348249 PMC4800740

[30] Barrington-Trimis JL, Urman R, Berhane K, E-cigarettes and future cigarette use. Pediatrics. 2016;138(1):e20160379. doi:10.1542/peds.2016-0379 27296866 PMC4925085

[31] Spindle TR, Hiler MM, Cooke ME, Eissenberg T, Kendler KS, Dick DM Electronic cigarette use and uptake of cigarette smoking: a longitudinal examination of U.S. college students. Addict Behav. 2017;67:66-72. doi:10.1016/j.addbeh.2016.12.009 28038364 PMC5250543

[32] Miech R, Patrick ME, O’Malley PM, Johnston LD E-cigarette use as a predictor of cigarette smoking: results from a 1-year follow-up of a national sample of 12th grade students. Tob Control. 2017;26(e2):e106-e111. doi:10.1136/tobaccocontrol-2016-053291 28167683 PMC5545162

[33] Loukas A, Marti CN, Cooper M, Pasch KE, Perry CL Exclusive e-cigarette use predicts cigarette initiation among college students. Addict Behav. 2018;76:343-347. doi:10.1016/j.addbeh.2017.08.023 28892771 PMC5614895

[34] Morgenstern M, Nies A, Goecke M, Hanewinkel R E-cigarettes and the use of conventional cigarettes. Dtsch Arztebl Int. 2018;115(14):243-248. doi:10.3238/arztebl.2018.0243 29716689 PMC5938547

[35] Bold KW, Kong G, Camenga DR, Trajectories of e-cigarette and conventional cigarette use among youth. Pediatrics. 2018;141(1):e20171832. doi:10.1542/peds.2017-1832 29203523 PMC5744268

[36] Hammond D, Reid JL, Cole AG, Leatherdale ST Electronic cigarette use and smoking initiation among youth: a longitudinal cohort study. CMAJ. 2017;189(43):E1328-E1336. doi:10.1503/cmaj.161002 29084759 PMC5662449

[37] Aleyan S, Cole A, Qian W, Leatherdale ST Risky business: a longitudinal study examining cigarette smoking initiation among susceptible and non-susceptible e-cigarette users in Canada. BMJ Open. 2018;8(5):e021080. doi:10.1136/bmjopen-2017-021080 29804064 PMC5988055

[38] Conner M, Grogan S, Simms-Ellis R, Do electronic cigarettes increase cigarette smoking in UK adolescents? evidence from a 12-month prospective study. Tob Control. 2017;27(4):365-372. doi:10.1136/tobaccocontrol-2016-053539 28818839 PMC6047139

[39] Treur JL, Rozema AD, Mathijssen JJP, van Oers H, Vink JM E-cigarette and waterpipe use in two adolescent cohorts: cross-sectional and longitudinal associations with conventional cigarette smoking. Eur J Epidemiol. 2018;33(3):323-334. doi:10.1007/s10654-017-0345-9 29260431 PMC5889768

[40] Lozano P, Barrientos-Gutierrez I, Arillo-Santillan E, A longitudinal study of electronic cigarette use and onset of conventional cigarette smoking and marijuana use among Mexican adolescents. Drug Alcohol Depend. 2017;180:427-430. doi:10.1016/j.drugalcdep.2017.09.001 28988005 PMC5771440

[41] Best C, Haseen F, Currie D, Relationship between trying an electronic cigarette and subsequent cigarette experimentation in Scottish adolescents: a cohort study. Tob Control. 2017;27(4):373-378. doi:10.1136/tobaccocontrol-2017-053691 28735273 PMC6047138

[42] Wills TA, Knight R, Sargent JD, Gibbons FX, Pagano I, Williams RJ Longitudinal study of e-cigarette use and onset of cigarette smoking among high school students in Hawaii. Tob Control. 2017;26(1):34-39. doi:10.1136/tobaccocontrol-2015-052705 26811353 PMC4959970

[43] Unger JB, Soto DW, Leventhal A E-cigarette use and subsequent cigarette and marijuana use among Hispanic young adults. Drug Alcohol Depend. 2016;163:261-264. doi:10.1016/j.drugalcdep.2016.04.027 27141841 PMC7453602

[44] Soneji S, Barrington-Trimis JL, Wills TA Errors in data input in meta-analysis on association between initial use of e-cigarettes and subsequent cigarette smoking among adolescents and young adults. Jama Pediatr. 2018;172(1):92-93. doi:10.1001/jamapediatrics.2017.4200 29131876

[45] Parsons VL, Moriarity CL, Jonas K, Moore TF, Davis KE, Tompkins L Design and estimation for the national health interview survey, 2006-2015. Vital Health Stat 2. 2014;165:1-53. 24775908

