# Supplementary material for: Trends in the Age of Cigarette Smoking Initiation Among Young Adults in the US From 2002 to 2018
Source: JAMA Netw Open. 2020 Oct 6;3(10):e2019022. doi: 10.1001/jamanetworkopen.2020.19022 (PMC7539122; doi:10.1001/jamanetworkopen.2020.19022)
Supplement: Supplement. — eAppendix. Supplemental Methods eTable 1. Demographic Characteristics of Participants Included in the Analytic Sample Among Those Aged 22-23 At Survey Completion, by Year, From 2002-2018 eTable 2. Cumulative Proportion of Participants Who Had Ever Initiated Cigarette Smoking, by Each Age of Initiation and Year (2002-2018), Among Ever Smokers and Among Ever Smokers Who Had Smoked More Than 100 Cigarettes, for Survey Respondents Aged 22-23 at Survey Completion eTable 3. Cumulative Proportion of Participants Who Had Ever Smoked Daily, by Each Age of First Daily Cigarette Smoking and Year (2002-2018), Among Ever Daily Smokers and Among Ever Daily Smokers Who Had Smoked More Than 100 Cigarettes for Survey Respondents Aged 22-23 at Survey Completion eFigure 1. Prevalence (95% CI) of Ever Smoking, and Daily Smoking by Year From 2002-2018, Among Survey Participants Aged 22-23 eFigure 2. Distribution of (A) Age at Initiation Among Ever Cigarette Smokers and (B) Age at Transition to Daily Smoking Among Ever Daily Smokers, by Year, Among Participants Aged 22-23 at Survey Administration eFigure 3. Proportion (95% CI) of Ever Cigarette Smokers Who Began Smoking at Age 18 or Older, and Proportion of Ever Daily Smokers Who Began Smoking Daily at Age 18 or Older, by Year From 2002-2018, Among Participants (A) Aged 21 and (B) Aged 24-25 at Survey Completion eFigure 4. Proportion (95% CI) of Regular Cigarette Smokers Who Began Smoking at Age 18 or Older in the NHIS Study, by Year From 2002-2018, Among Participants Aged 22-23 at Survey Completion [file jamanetwopen-e2019022-s001.pdf]

Supplemental Online Content

Barrington-Trimis JL, Braymiller JL, Unger JB. Trends in the age of cigarette smoking initiation among young adults in the US from 2002 to 2018. *JAMA Netw Open*. 2020;3(10):e2019022. doi:10.1001/jamanetworkopen.2020.19022

eAppendix. Supplemental Methods
eTable 1. Demographic Characteristics of Participants Included in the Analytic Sample Among Those Aged 22-23 At Survey Completion, by Year, From 2002-2018
eTable 2. Cumulative Proportion of Participants Who Had Ever Initiated Cigarette Smoking, by Each Age of Initiation and Year (2002-2018), Among Ever Smokers and Among Ever Smokers Who Had Smoked More Than 100 Cigarettes, for Survey Respondents Aged 22-23 at Survey Completion
eTable 3. Cumulative Proportion of Participants Who Had Ever Smoked Daily, by Each Age of First Daily Cigarette Smoking and Year (2002-2018), Among Ever Daily Smokers and Among Ever Daily Smokers Who Had Smoked More Than 100 Cigarettes for Survey Respondents Aged 22-23 at Survey Completion
eFigure 1. Prevalence (95% CI) of Ever Smoking, and Daily Smoking by Year From 2002-2018, Among Survey Participants Aged 22-23
eFigure 2. Distribution of (A) Age at Initiation Among Ever Cigarette Smokers and (B) Age at Transition to Daily Smoking Among Ever Daily Smokers, by Year, Among Participants Aged 22-23 at Survey Administration
eFigure 3. Proportion (95% CI) of Ever Cigarette Smokers Who Began Smoking at Age 18 or Older, and Proportion of Ever Daily Smokers Who Began Smoking Daily at Age 18 or Older, by Year From 2002-2018, Among Participants (A) Aged 21 and (B) Aged 24-25 at Survey Completion
eFigure 4. Proportion (95% CI) of Regular Cigarette Smokers Who Began Smoking at Age 18 or Older in the NHIS Study, by Year From 2002-2018, Among Participants Aged 22-23 at Survey Completion

This supplemental material has been provided by the authors to give readers additional information about their work.

© 2020 Barrington-Trimis J et al. *JAMA Network Open.*

eAppendix. Supplemental Methods

Participants

National Health Interview Survey (NHIS). We aimed to replicate results from NSDUH in a second dataset that utilized slightly different definitions of established (regular) smoking. Analyses were restricted to participants who completed the NHIS at any year between 2002 and 2018, who were 22 or 23 at the time of survey, and who met the definition for having ever been a regular cigarette smoker (see measures below) (N=4,394). Data collection procedures have been described elsewhere.[46] Briefly, the NHIS is an annual survey in the U.S. that aims to monitor "health status, health care access, and progress toward achieving national health objectives" (https://www.cdc.gov/nchs/nhis/index.htm) NHIS also uses a multi-stage, stratified, complex clustering methodology and is representative of the US population, with data also collected using computer assisted personal interviewing methods.

Measures

Regular smoking. In the NHIS, participants were considered ever smokers if they had smoked at least 100 cigarettes in their lifetime; ever smokers were asked the age at which they had first smoked cigarettes regularly (age of regular smoking initiation, in years).

Sociodemographic characteristics. Participants reported gender (male or female), race/ethnicity (Non-Hispanic [NH] White, NH Black/African American, NH Native American/Alaska Native, NH Asian, NH multiple race/other/unknown, Hispanic), highest level of education (some high school or less, high school diploma or GED, some college, college degree or higher), and marital status (never married, married, divorced or separated, living with partner, other).

© 2020 Barrington-Trimis J et al. *JAMA Network Open.*

eTable 1. Demographic Characteristics of Participants Included in the Analytic Sample Among Those Aged 22-23 at Survey Completion, by Year, From 2002-2018

	Survey Year																
	2002	2003	2004	2005	2006	2007	2008	2009	2010	2011	2012	2013	2014	2015	2016	2017	2018
Gender																	
Male	50.9%	48.9%	49.9%	49.8%	49.9%	49.2%	50.0%	49.8%	50.0%	49.4%	48.5%	50.1%	50.7%	49.2%	47.7%	48.3%	49.0%
Female	49.1%	51.1%	50.1%	50.2%	50.1%	50.8%	50.0%	50.2%	50.0%	50.6%	51.5%	49.9%	49.3%	50.8%	52.3%	51.7%	51.0%
Race/Ethnicity																	
NH White	60.7%	62.2%	61.7%	62.9%	63.0%	60.1%	62.6%	60.9%	61.0%	57.1%	58.0%	56.2%	56.8%	56.3%	54.7%	54.2%	55.6%
NH Black	12.7%	12.7%	13.4%	13.5%	13.2%	14.5%	13.0%	14.7%	13.7%	14.6%	14.3%	14.8%	14.5%	13.9%	13.6%	13.9%	13.4%
NH Nat. Amer./Alaska Nat.	0.8%	0.5%	0.4%	0.7%	0.4%	0.7%	0.5%	0.7%	0.6%	0.5%	0.9%	0.9%	0.6%	0.7%	1.0%	0.7%	0.8%
NH Hawaiian/Pac. Is.	0.4%	0.4%	0.4%	0.5%	0.6%	0.3%	0.3%	0.3%	0.5%	0.4%	0.5%	0.6%	0.5%	0.6%	0.6%	0.4%	0.4%
NH Asian	5.1%	4.9%	4.7%	4.0%	4.0%	5.2%	4.5%	4.4%	4.4%	5.2%	5.1%	5.0%	6.2%	6.1%	7.0%	6.5%	6.5%
NH Multiple Races	1.2%	1.1%	1.2%	1.0%	1.2%	1.4%	1.1%	1.2%	1.5%	1.7%	1.6%	1.6%	2.1%	2.1%	2.4%	2.2%	1.9%
Hispanic	19.0%	18.1%	18.2%	17.4%	17.5%	17.9%	17.9%	17.7%	18.3%	20.5%	19.4%	20.9%	19.3%	20.4%	20.7%	22.1%	21.4%
Highest Level of Education																	
Less than high school	18.3%	15.7%	18.5%	15.5%	15.8%	13.9%	13.2%	13.4%	11.2%	12.0%	10.4%	11.4%	9.4%	10.2%	7.1%	7.1%	8.6%
High school diploma/GED	28.8%	29.2%	27.9%	29.5%	29.3%	28.7%	29.7%	28.4%	28.0%	29.3%	28.5%	29.3%	27.4%	25.8%	27.2%	27.8%	24.2%
Some college	31.3%	32.0%	33.0%	32.0%	31.4%	33.7%	31.9%	33.2%	34.8%	33.6%	35.0%	33.8%	37.2%	44.1%	43.0%	40.7%	41.0%
College degree or higher	21.6%	23.1%	20.6%	22.9%	23.6%	23.7%	25.3%	24.9%	26.0%	25.0%	26.1%	25.5%	26.0%	19.9%	22.7%	24.4%	26.2%
Annual Family Income																	
Less than $20,000	34.2%	34.9%	33.3%	31.9%	33.2%	30.9%	31.4%	32.5%	34.1%	33.6%	31.2%	33.3%	31.8%	30.8%	28.6%	27.5%	26.6%
$20,000-$49,999	41.2%	38.7%	39.7%	38.6%	37.9%	35.9%	36.7%	36.6%	34.0%	34.5%	34.9%	36.5%	33.7%	34.8%	33.4%	33.6%	35.1%
$50,000-$74,999	12.2%	12.0%	12.7%	11.6%	12.0%	14.9%	14.0%	11.5%	13.9%	12.2%	13.2%	12.1%	14.0%	12.3%	13.7%	13.5%	12.6%
$75,000 or higher	12.5%	14.4%	14.3%	17.9%	16.9%	18.3%	17.8%	19.4%	18.0%	19.7%	20.7%	18.1%	20.5%	22.1%	24.3%	25.4%	25.7%
Marital Status																	
Never married	75.8%	78.6%	77.5%	79.9%	81.0%	81.7%	82.3%	82.6%	83.5%	85.9%	86.6%	86.2%	87.0%	85.5%	87.5%	88.3%	87.2%
Married	21.0%	18.6%	19.9%	17.0%	16.8%	15.6%	15.5%	15.1%	14.5%	12.3%	11.8%	11.7%	11.6%	11.5%	10.9%	10.4%	11.4%
Divorced or Separated	3.0%	2.6%	2.5%	2.9%	2.0%	2.7%	2.2%	2.2%	2.0%	1.6%	1.6%	2.0%	1.4%	1.6%	1.5%	1.3%	1.4%
Widowed	0.1%	0.2%	0.2%	0.2%	0.2%	0.1%	0.0%	0.1%	0.0%	0.1%	0.1%	0.1%	0.0%	1.5%	0.1%	0.0%	0.0%

© 2020 Barrington-Trimis J et al. *JAMA Network Open.*

eTable 2. Cumulative Proportion of Participants Who Had Ever Initiated Cigarette Smoking, by Each Age of Initiation and Year (2002-2018), Among Ever Smokers and Among Ever Smokers Who Had Smoked More Than 100 Cigarettes, for Survey Respondents Aged 22-23 at Survey Completion

Age	2002	2003	2004	2005	2006	2007	2008	2009	2010	2011	2012	2013	2014	2015	2016	2017	2018
Among ever smokers																	
	N=3310	N=3372	N=3297	N=3238	N=3166	N=3104	N=3136	N=3129	N=3111	N=3126	N=3057	N=2952	N=2083	N=2154	N=1953	N=2009	N=1818
<10	4.5%	3.8%	3.7%	3.9%	4.5%	4.3%	3.4%	3.1%	2.6%	2.9%	2.7%	3.0%	3.1%	2.5%	1.9%	1.9%	2.2%
<11	7.1%	6.8%	6.7%	6.5%	7.0%	7.0%	5.7%	5.1%	4.4%	5.0%	4.2%	5.2%	5.2%	3.9%	3.1%	3.1%	2.9%
<12	9.6%	9.8%	8.7%	9.5%	10.3%	10.6%	8.3%	7.8%	6.1%	6.7%	5.6%	7.2%	6.2%	5.3%	4.6%	4.3%	4.0%
<13	20.8%	18.7%	19.7%	20.0%	20.6%	19.7%	17.3%	17.5%	12.8%	12.3%	12.0%	12.5%	11.2%	10.0%	9.4%	9.6%	8.1%
<14	30.8%	30.9%	31.0%	31.6%	32.2%	29.6%	27.2%	26.1%	20.1%	20.7%	18.5%	19.9%	18.6%	15.9%	15.6%	16.0%	14.2%
<15	42.1%	43.2%	43.7%	43.2%	43.8%	39.7%	38.7%	35.8%	29.6%	29.0%	26.7%	26.1%	26.6%	24.5%	24.6%	23.8%	23.4%
<16	56.3%	56.2%	58.3%	56.4%	57.7%	53.6%	50.3%	48.6%	41.5%	40.6%	38.3%	35.3%	38.1%	37.7%	35.9%	34.6%	35.2%
<17	70.5%	69.7%	70.3%	70.6%	70.1%	65.7%	63.7%	61.8%	56.7%	53.9%	52.2%	50.1%	51.7%	50.4%	47.8%	48.4%	49.1%
<18	79.4%	78.4%	79.4%	79.3%	77.4%	74.1%	72.3%	70.9%	66.5%	63.6%	64.1%	61.3%	62.3%	61.2%	60.7%	58.7%	57.4%
<19	88.9%	89.2%	89.1%	88.4%	87.7%	86.9%	85.7%	85.5%	82.6%	80.0%	80.6%	80.7%	80.2%	79.3%	79.9%	77.6%	75.7%
<20	92.7%	93.6%	93.0%	93.9%	92.5%	91.3%	91.3%	90.9%	90.0%	88.6%	87.6%	88.6%	86.7%	87.5%	87.4%	85.7%	85.1%
<21	96.8%	97.0%	96.3%	96.8%	96.6%	95.2%	95.8%	95.9%	95.3%	94.5%	93.8%	95.2%	93.6%	94.1%	92.4%	92.9%	91.7%
<22	99.2%	99.1%	98.2%	98.7%	98.9%	97.9%	98.2%	98.4%	97.7%	98.4%	98.3%	98.6%	97.8%	97.7%	98.6%	97.6%	96.9%
<23	99.8%	99.8%	99.6%	99.6%	99.5%	99.8%	99.7%	99.6%	99.6%	99.6%	99.7%	99.7%	99.6%	99.6%	99.4%	99.7%	99.7%
Among ever smokers who had smoked more than 100 cigarettes																	
	N=2054	N=2091	N=2090	N=2014	N=1938	N=1861	N=1831	N=1808	N=1737	N=1714	N=1664	N=1611	N=1062	N=1086	N=951	N=958	N=880
<10	5.3%	4.6%	4.9%	4.7%	5.5%	5.3%	4.0%	3.5%	3.7%	3.8%	3.4%	3.7%	4.3%	3.0%	2.0%	2.6%	2.8%
<11	8.2%	8.1%	8.9%	8.3%	8.7%	8.7%	7.0%	6.3%	6.3%	6.5%	5.3%	6.6%	7.5%	4.5%	3.5%	4.2%	3.7%
<12	11.6%	12.1%	11.6%	12.4%	12.9%	13.2%	11.0%	9.6%	9.1%	8.8%	7.3%	9.3%	8.9%	6.7%	6.4%	6.1%	5.7%
<13	25.5%	23.7%	24.6%	25.5%	25.0%	25.1%	22.7%	22.3%	17.9%	15.8%	16.3%	16.7%	15.8%	13.7%	13.8%	14.9%	11.2%
<14	37.5%	36.9%	38.5%	38.6%	39.1%	36.5%	34.4%	33.0%	27.4%	27.3%	25.2%	27.5%	27.4%	21.0%	23.3%	24.8%	19.8%
<15	50.9%	51.7%	52.6%	52.6%	51.9%	47.1%	47.1%	45.0%	40.0%	38.8%	35.9%	35.8%	37.8%	32.5%	36.8%	35.5%	34.0%
<16	67.1%	67.4%	68.1%	68.0%	67.2%	62.3%	61.2%	59.8%	53.6%	53.2%	51.6%	47.5%	51.9%	50.1%	52.2%	51.0%	49.7%
<17	80.0%	80.3%	80.1%	81.2%	79.4%	74.8%	73.9%	73.8%	70.8%	69.2%	67.8%	64.2%	66.8%	65.1%	66.6%	67.4%	65.5%
<18	87.9%	87.2%	88.2%	88.5%	85.5%	82.4%	81.5%	81.4%	80.3%	77.7%	78.8%	76.4%	77.1%	74.4%	78.7%	78.6%	74.1%
<19	94.9%	94.4%	95.2%	95.4%	93.1%	93.0%	92.8%	93.5%	91.6%	90.7%	90.6%	92.3%	90.9%	90.7%	92.7%	92.3%	90.6%
<20	96.9%	97.3%	97.5%	98.0%	97.0%	96.1%	96.6%	96.7%	97.1%	96.1%	95.2%	95.5%	93.9%	94.6%	96.4%	95.9%	95.6%
<21	99.1%	98.6%	99.0%	99.0%	98.9%	98.2%	98.9%	98.9%	98.6%	98.6%	98.2%	98.4%	98.1%	97.8%	98.6%	98.2%	98.6%
<22	99.8%	99.5%	99.6%	99.8%	99.7%	99.0%	99.6%	99.6%	99.1%	99.8%	99.6%	99.6%	99.2%	99.5%	99.9%	99.7%	99.7%
<23	100%	99.9%	100%	100%	99.9%	100%	100%	99.8%	100%	100%	100%	100%	100%	99.9%	100%	100%	100%

© 2020 Barrington-Trimis J et al. *JAMA Network Open.*

eTable 3. Cumulative Proportion of Participants Who Had Ever Smoked Daily, by Each Age of First Daily Cigarette Smoking and Year (2002-2018), Among Ever Daily Smokers and Among Ever Daily Smokers Who Had Smoked More Than 100 Cigarettes for Survey Respondents Aged 22-23 at Survey Completion.

Age	2002	2003	2004	2005	2006	2007	2008	2009	2010	2011	2012	2013	2014	2015	2016	2017	2018
Among ever daily smokers																	
	N=1866	N=1886	N=1873	N=1825	N=1757	N=1683	N=1650	N=1615	N=1570	N=1516	N=1502	N=1431	N=961	N=942	N=825	N=830	N=758
<10	0.8%	0.5%	0.3%	0.5%	0.6%	0.8%	0.8%	0.3%	0.3%	0.3%	1.2%	0.2%	0.6%	0.0%	0.3%	0.2%	0.2%
<11	1.5%	0.8%	1.1%	0.8%	1.6%	1.4%	1.3%	0.5%	0.6%	1.0%	1.6%	1.1%	1.2%	0.8%	0.7%	0.8%	0.5%
<12	2.7%	1.6%	1.9%	1.8%	2.6%	2.0%	2.0%	1.0%	1.2%	1.5%	2.3%	1.8%	2.1%	1.4%	1.0%	1.1%	1.3%
<13	6.0%	3.9%	4.6%	5.0%	6.1%	4.3%	5.9%	3.0%	3.6%	3.1%	4.7%	3.8%	3.6%	3.4%	2.6%	3.3%	3.4%
<14	11.7%	9.2%	10.6%	10.6%	11.0%	10.1%	11.0%	7.8%	7.5%	6.0%	8.2%	7.9%	7.1%	6.7%	4.9%	7.8%	6.0%
<15	19.2%	16.8%	18.6%	16.3%	18.3%	17.6%	18.0%	13.5%	13.5%	11.9%	13.8%	11.8%	11.2%	11.4%	10.2%	13.5%	11.0%
<16	30.3%	29.9%	30.5%	29.6%	29.8%	29.8%	28.6%	25.3%	21.2%	20.1%	22.8%	21.0%	20.8%	20.4%	20.4%	20.6%	19.1%
<17	47.9%	51.0%	47.6%	47.4%	45.0%	45.6%	44.2%	39.9%	35.4%	34.7%	35.5%	35.9%	33.8%	34.8%	36.7%	34.4%	32.0%
<18	61.3%	62.7%	61.2%	60.9%	59.5%	58.4%	56.9%	53.7%	51.6%	45.8%	48.8%	46.6%	47.2%	47.1%	49.3%	48.4%	44.1%
<19	79.9%	80.2%	80.6%	80.5%	77.2%	76.0%	76.5%	73.5%	75.0%	70.3%	72.3%	70.5%	72.6%	72.1%	74.3%	74.3%	66.9%
<20	89.4%	86.5%	89.2%	88.0%	87.5%	86.8%	85.0%	83.9%	84.8%	82.4%	82.5%	82.5%	82.6%	81.7%	84.0%	85.9%	78.0%
<21	95.3%	94.2%	95.2%	94.8%	93.8%	93.7%	93.2%	92.6%	93.9%	91.8%	91.7%	91.2%	91.1%	89.7%	93.5%	94.5%	92.3%
<22	98.6%	98.3%	97.9%	98.6%	97.8%	97.9%	98.0%	97.9%	97.3%	97.8%	97.1%	97.5%	97.5%	95.6%	98.2%	97.7%	97.8%
<23	99.9%	99.4%	99.2%	99.7%	99.1%	99.8%	99.8%	99.6%	99.6%	99.6%	99.6%	99.8%	99.7%	98.6%	99.5%	99.7%	99.5%
Among ever daily smokers who had smoked more than 100 cigarettes																	
	N=1807	N=1835	N=1825	N=1755	N=1692	N=1622	N=1589	N=1542	N=1507	N=1450	N=1436	N=1379	N=911	N=894	N=786	N=793	N=717
<10	0.9%	0.5%	0.3%	0.5%	0.6%	0.8%	0.6%	0.2%	0.3%	0.3%	1.2%	0.2%	0.6%	0.0%	0.1%	0.2%	0.2%
<11	1.6%	0.8%	1.1%	0.9%	1.7%	1.5%	1.2%	0.4%	0.6%	1.1%	1.7%	1.1%	1.3%	0.9%	0.5%	0.7%	0.5%
<12	2.8%	1.5%	1.9%	1.9%	2.7%	2.0%	1.8%	0.9%	1.3%	1.5%	2.4%	1.8%	2.2%	1.4%	0.8%	1.1%	1.4%
<13	6.2%	3.9%	4.7%	5.1%	6.3%	4.4%	5.7%	3.0%	3.6%	3.2%	4.9%	4.0%	3.7%	3.6%	2.4%	3.3%	3.6%
<14	12.0%	9.2%	10.8%	10.8%	11.4%	10.4%	10.9%	7.7%	7.6%	6.1%	8.4%	8.0%	7.3%	6.9%	4.9%	7.6%	6.2%
<15	19.5%	16.8%	18.9%	16.7%	18.5%	18.2%	18.0%	13.6%	13.8%	12.0%	13.9%	12.0%	11.4%	11.5%	10.2%	13.0%	11.5%
<16	30.8%	30.1%	30.8%	30.2%	30.2%	30.6%	28.7%	25.8%	21.6%	20.5%	23.4%	21.3%	21.0%	20.8%	20.7%	20.2%	19.8%
<17	48.7%	51.5%	48.1%	48.1%	45.7%	46.1%	44.8%	40.9%	35.9%	35.7%	36.3%	36.6%	34.0%	35.8%	37.1%	34.1%	33.3%
<18	62.2%	63.5%	61.5%	61.5%	60.6%	59.4%	57.6%	55.1%	52.5%	46.8%	50.2%	47.4%	47.6%	48.1%	50.1%	48.9%	45.5%
<19	80.8%	81.0%	81.2%	80.8%	77.7%	76.9%	77.0%	74.6%	76.2%	70.5%	73.5%	70.8%	73.1%	72.4%	75.4%	75.4%	68.3%
<20	90.3%	87.0%	89.7%	88.5%	88.0%	87.4%	85.6%	84.5%	85.9%	82.9%	83.4%	82.9%	82.5%	82.5%	84.9%	86.6%	79.6%
<21	95.8%	94.6%	95.7%	95.0%	93.8%	94.1%	93.7%	93.1%	94.3%	92.2%	92.8%	91.7%	91.3%	90.0%	94.4%	94.8%	93.6%
<22	98.8%	98.4%	98.1%	98.7%	97.8%	98.2%	98.2%	97.9%	97.6%	98.3%	97.6%	97.7%	98.0%	96.0%	98.8%	98.2%	98.5%
<23	99.9%	99.4%	99.3%	99.7%	99.1%	99.8%	99.8%	99.7%	99.7%	99.8%	99.6%	99.8%	99.8%	98.6%	99.7%	99.7%	99.9%

© 2020 Barrington-Trimis J et al. *JAMA Network Open.*

eFigure 1. Prevalence (95% CI) of Ever Smoking, and Daily Smoking by Year From 2002-2018, Among Survey Participants Aged 22-23

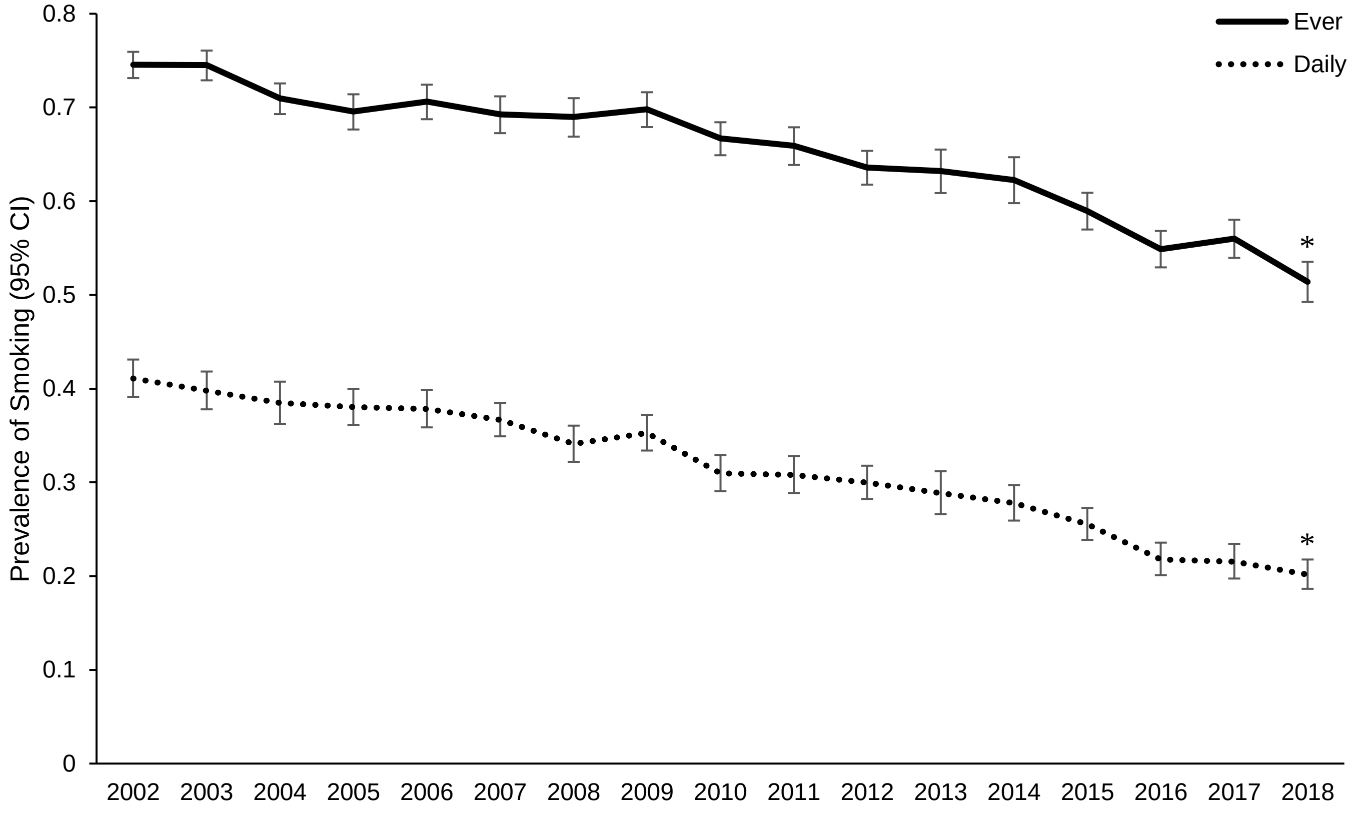

**P-value < 0.001 for test of difference in prevalence of smoking in 2002 vs. 2018, adjusting for gender, race/ethnicity, education level, income, and marital status*

© 2020 Barrington-Trimis J et al. *JAMA Network Open.*

eFigure 2. Distribution of (A) Age at Initiation Among Ever Cigarette Smokers and (B) Age at Transition to Daily Smoking Among Ever Daily Smokers, by Year, Among Participants Aged 22-23 at Survey Administration*

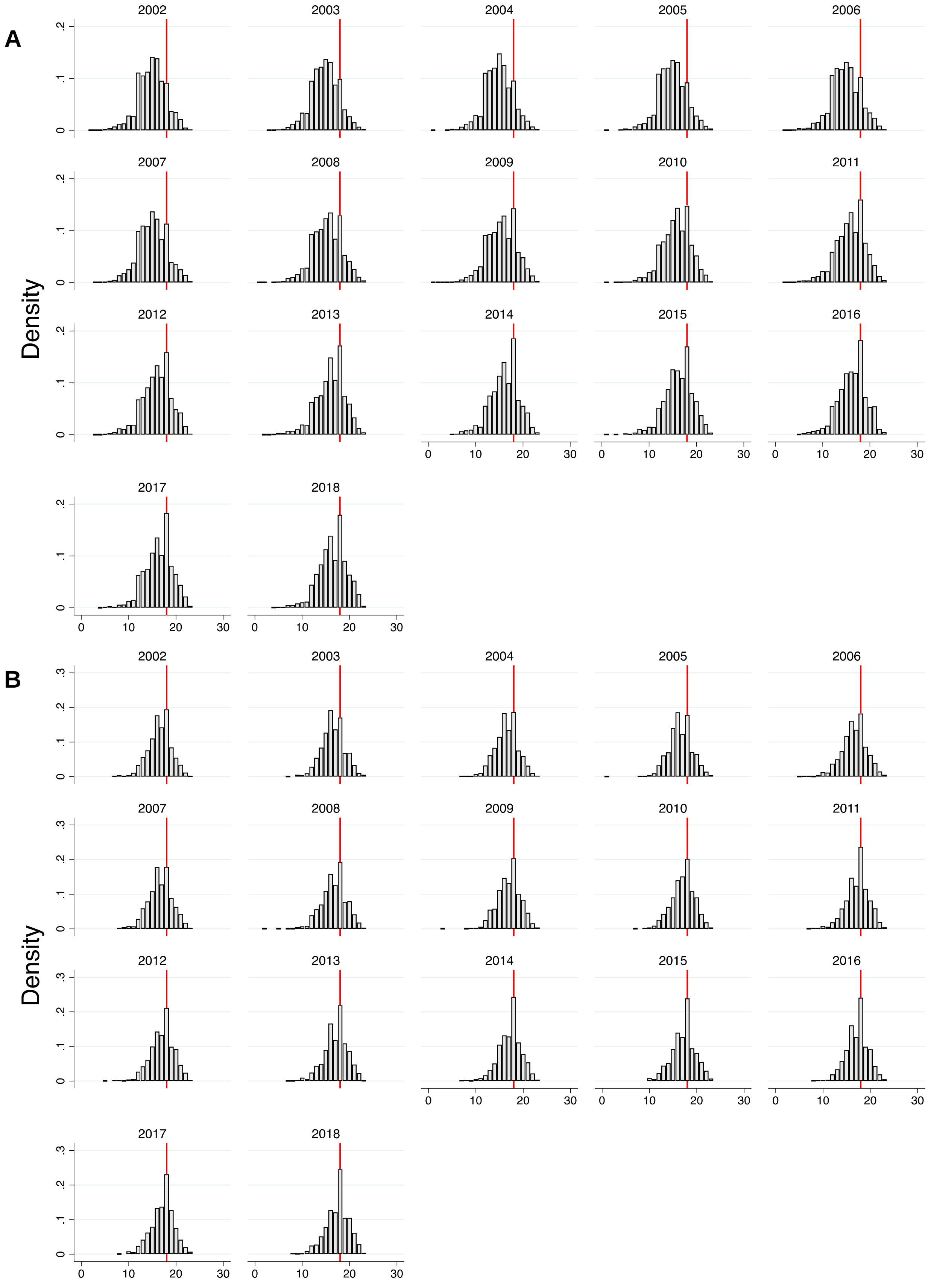

© 2020 Barrington-Trimis J et al. *JAMA Network Open.*

eFigure 3. Proportion (95% CI) of Ever Cigarette Smokers Who Began Smoking at Age 18 or Older, and Proportion of Ever Daily Smokers Who Began Smoking Daily at Age 18 or Older, by Year From 2002-2018, Among Participants (A) Aged 21 and (B) Aged 24-25 at Survey Completion.

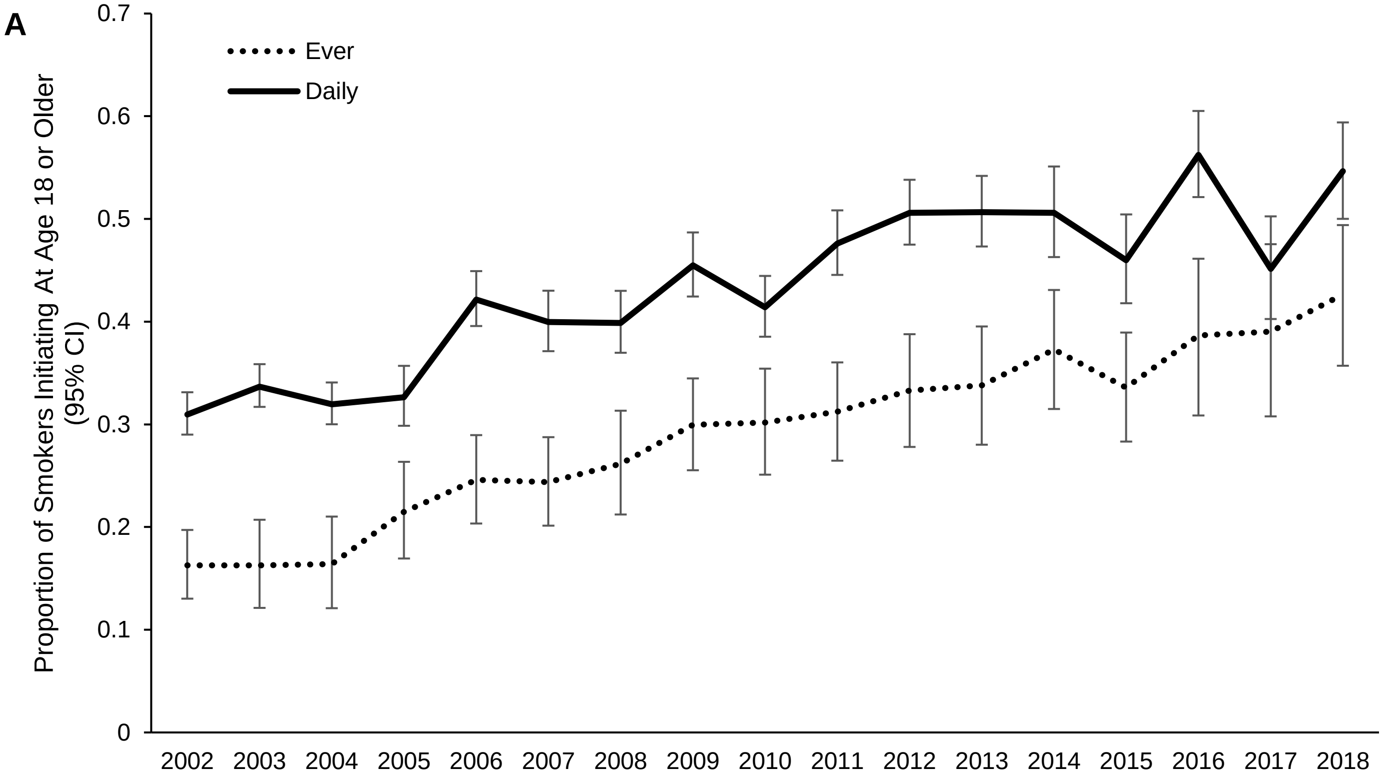

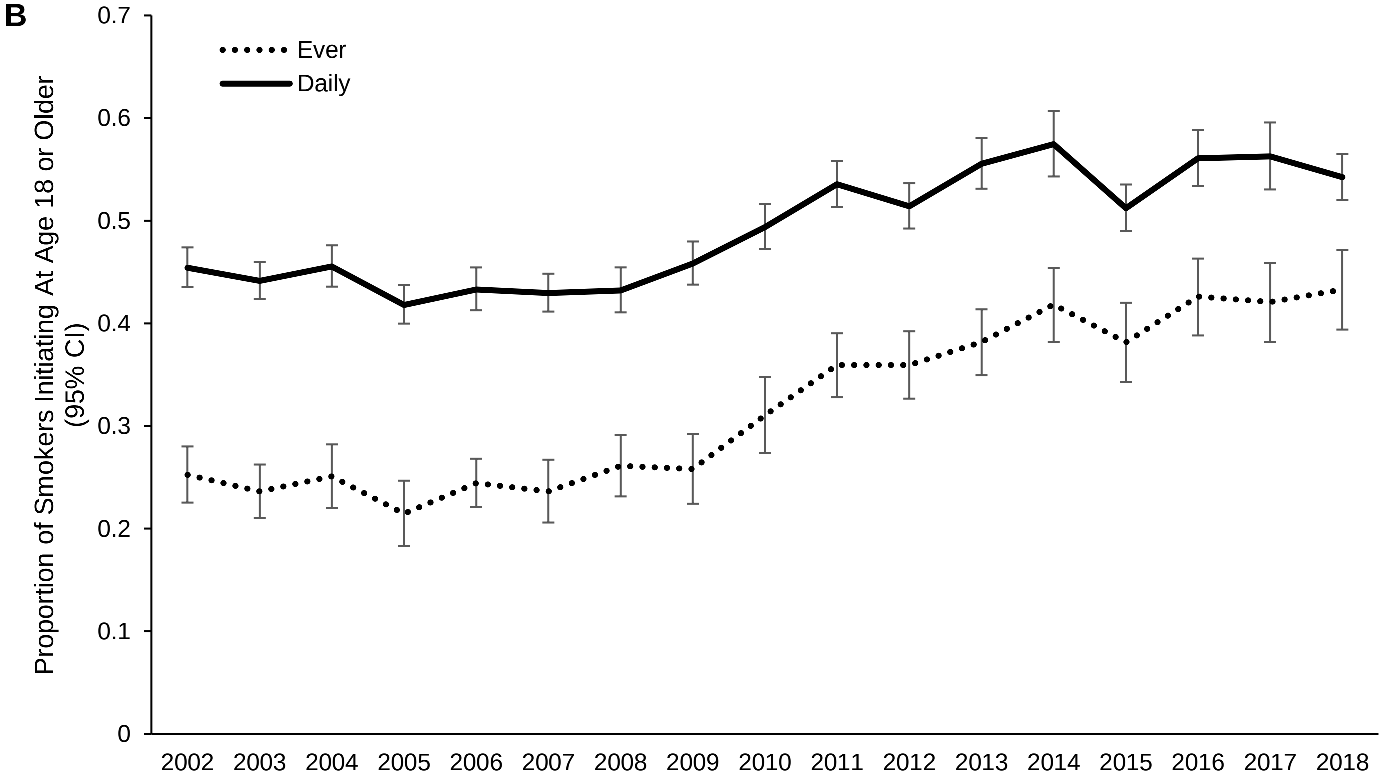

© 2020 Barrington-Trimis J et al. *JAMA Network Open.*

eFigure 4. Proportion (95% CI) of Regular Cigarette Smokers Who Began Smoking at Age 18 or Older in the NHIS Study, by Year From 2002-2018, Among Participants Aged 22-23 at Survey Completion.

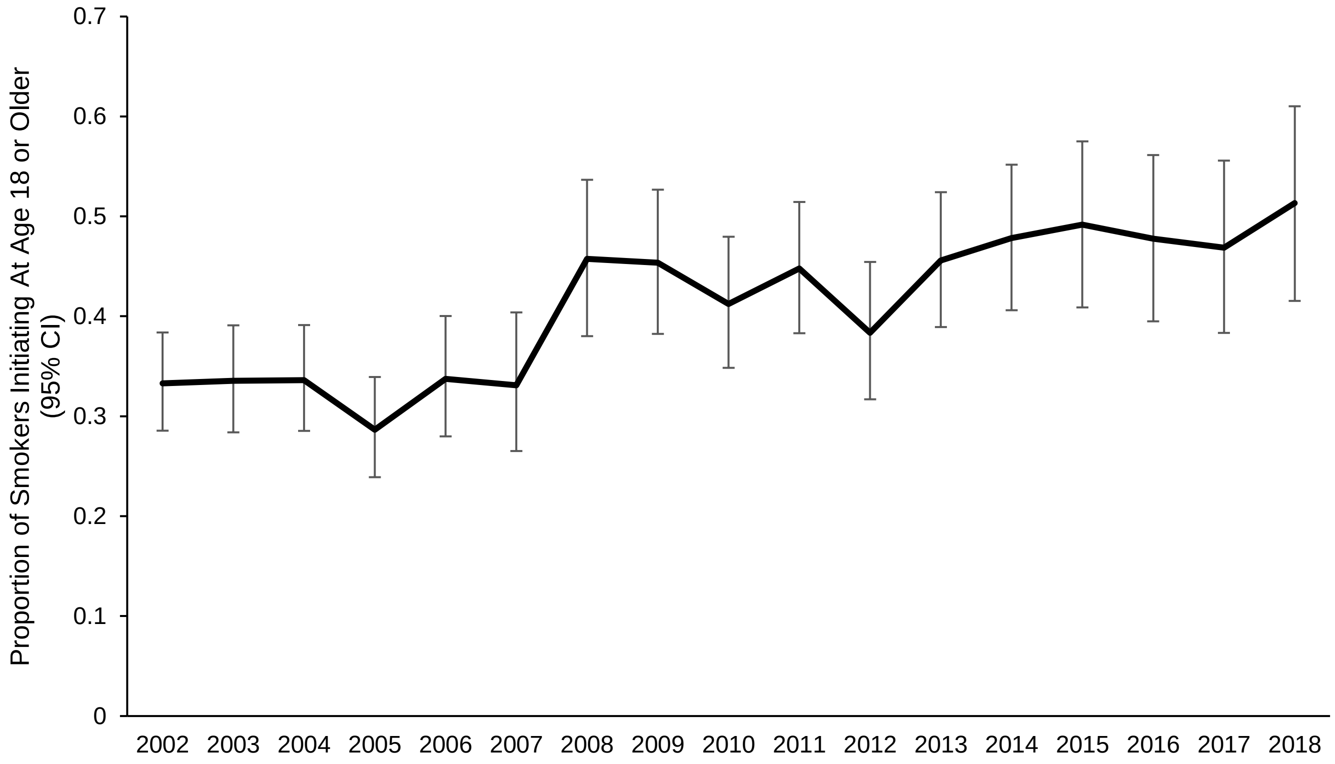

© 2020 Barrington-Trimis J et al. *JAMA Network Open.*
